# Residual malaria transmission and the role of *Anopheles arabiensis* and *Anopheles melas* in central Senegal

**DOI:** 10.1093/jme/tjad020

**Published:** 2023-03-18

**Authors:** Ousmane Sy, Pape C Sarr, Benoit S Assogba, Mouhamed A Nourdine, Assane Ndiaye, Lassana Konaté, Ousmane Faye, Martin J Donnelly, Oumar Gaye, David Weetman, Elhadji A Niang

**Affiliations:** Laboratoire d’Écologie Vectorielle et Parasitaire, Faculté des Sciences et Techniques, Université Cheikh Anta DIOP Dakar/Sénégal; Laboratory of Medical Parasitology (MARCAD program) Faculty of Medicine, Pharmacy and Odontostomatology of Cheikh Anta DIOP University of Dakar/Senegal; Laboratoire d’Écologie Vectorielle et Parasitaire, Faculté des Sciences et Techniques, Université Cheikh Anta DIOP Dakar/Sénégal; Laboratory of Medical Parasitology (MARCAD program) Faculty of Medicine, Pharmacy and Odontostomatology of Cheikh Anta DIOP University of Dakar/Senegal; Disease Control and Elimination Theme, Medical Research Council Unit, The Gambia at the London School of Hygiene and Tropical Medicine, Banjul P.O. Box 273, The Gambia; Laboratoire d’Écologie Vectorielle et Parasitaire, Faculté des Sciences et Techniques, Université Cheikh Anta DIOP Dakar/Sénégal; Laboratory of Medical Parasitology (MARCAD program) Faculty of Medicine, Pharmacy and Odontostomatology of Cheikh Anta DIOP University of Dakar/Senegal; Laboratoire d’Écologie Vectorielle et Parasitaire, Faculté des Sciences et Techniques, Université Cheikh Anta DIOP Dakar/Sénégal; Laboratoire d’Écologie Vectorielle et Parasitaire, Faculté des Sciences et Techniques, Université Cheikh Anta DIOP Dakar/Sénégal; Laboratoire d’Écologie Vectorielle et Parasitaire, Faculté des Sciences et Techniques, Université Cheikh Anta DIOP Dakar/Sénégal; Department of Vector Biology, Liverpool School of Tropical Medicine, Pembroke Place, Liverpool L3 5QA, United Kingdom; Laboratory of Medical Parasitology (MARCAD program) Faculty of Medicine, Pharmacy and Odontostomatology of Cheikh Anta DIOP University of Dakar/Senegal; Department of Vector Biology, Liverpool School of Tropical Medicine, Pembroke Place, Liverpool L3 5QA, United Kingdom; Laboratoire d’Écologie Vectorielle et Parasitaire, Faculté des Sciences et Techniques, Université Cheikh Anta DIOP Dakar/Sénégal

**Keywords:** hotspots, malaria, elimination, qPCR, Senegal

## Abstract

Understanding the behavior and ecology of local malaria vectors is essential for the effectiveness of the commonly used vector-targeted malaria control tools in areas of low malaria transmission. This study was conducted to determine species composition, biting behavior and infectivity of the major *Anopheles* vectors of *Plasmodium falciparum* in low transmission settings in central Senegal. Adult mosquitoes were collected using human landing catches during 2 consecutive nights and Pyrethrum Spray Catches in 30–40 randomly selected rooms, from July 2017 to December 2018 in 3 villages. Anopheline mosquitoes were morphologically identified using conventional keys; their reproductive status assessed by ovary dissections, and a sub-sample of *Anopheles gambiae* s.l. were identified to species level using polymerase chain reaction (PCR). *Plasmodium* sporozoite infections were detected using real-time quantitative PCR. During this study 3684 *Anopheles* were collected of which 97% were *An. gambiae* s.l., 0.6% were *Anopheles funestus*, and 2.4% were *Anopheles pharoensis*. Molecular identification of 1,877 *An. gambiae* s.l. revealed a predominance of *Anopheles arabiensis* (68.7%), followed by *Anopheles melas* (28.8%), and *Anopheles coluzzii* (2.1%). The overall human-biting rate of *An. gambiae* s.l. was highest in the inland site of Keur Martin with 4.92 bites per person per night, while it was similar in the deltaic site, Diofior (0.51) and the coastal site, Mbine Coly (0.67). Parity rates were similar in *An. arabiensis* (45%) and *An. melas* (42%). Sporozoite infections were detected in both *An. arabiensis* and *An. melas* with the respective infection rates of 1.39% (*N* = 8) and 0.41% (*N* = 1). Results suggest that low residual malaria in central Senegal is transmitted by *An. arabiensis* and *An. melas*. Consequently, both vectors will need to be targeted as part of malaria elimination efforts in this area of Senegal.

## Introduction

Malaria remains one of the most important parasitic diseases worldwide. According to WHO, 241 million malaria cases and 627,000 deaths were reported worldwide in 2020, representing increases of 6% and 12%, respectively, compared to 2019 (WHO 2021, OMS 2019). Service disruptions during the COVID-19 pandemic are likely to have contributed to these increases, but a stagnation in malaria declines was also observed in many parts of Sub-Saharan Africa prior to the pandemic (WHO 2021). In contrast, between 2015 and 2019, Senegal recorded a 38% decrease in the number of malaria cases (from 69 to 50 per 1,000 population) and a 7% decrease in malaria deaths (from 0.30 to 0.28 per 1,000 population) (WHO 2020) and is among the countries displaying the lowest malaria incidence in the Western African region (PMI 2018, PNLP 2018).

Malaria transmission is typically more intense where the anopheline species have marked preference for humans and live long enough to allow completion of parasite sporogony development. The lifespan of African anopheline species and their highly anthropophilic behavior are among the reasons that the continent bears the highest malaria burden worldwide (Fontenille and Lochouarn 1999). Across Sub-Saharan Africa, the primary malaria vectors belong to the *Anopheles gambiae* sensu lato (s.l.) complex and *Anopheles funestus* s.l. group, targeting of which by vector control interventions is considered crucial for malaria control and elimination. In Senegal, 22 anopheline species have been reported (Diagne et al. 1994, Coetzee et al. 2000; Niang et al. 2014), of which 4 are *An. gambiae* s.l. (*Anopheles arabiensis, Anopheles melas, An. gambiae*, and *An. coluzzi*), which along with *An. funestus* s.s. are reported as the primary malaria vectors (Vercruysse and Jancloes 1981; Gazin and Robert 1987; Robert et al. 1998, Konate et al. 1994; Lemasson et al. 1997; Coetzee et al. 2000; Niang et al. 2016; Sy et al. 2018).

In the central-western part of Senegal, the implementation of several control interventions, including seasonal malaria chemoprevention (SMC) from 2008 to 2011 followed by community-based indoor residual spraying with pirimiphos-methyl between 2013 and 2014, and universal LLIN distributions, have contributed to substantially reduce malaria burden in the area (Cissé et al. 2016, WHO/GMP 2011, Sy et al. 2019). However, despite the success recorded at the regional administrative level, a few residual transmission hotspot areas persist where especially suitable micro-environmental conditions for vector development occur (Ndiaye et al. 2020). Recent investigations in central Senegal reported the presence of *An. arabiensis*, *Anopheles coluzzii*, and *An. melas* in the hotspots surveyed (Sy et al. 2018), with local populations displaying resistance to several public health insecticides (Sy et al. 2021).

This longitudinal entomological study took place between July 2017 and December 2018 in 3 mains ecological (coastal, deltaic, and inland) settings in central Senegal to clarify the potential role and relative contributions of local populations of *An. arabiensis* and *An. melas* in maintaining malaria transmission in local hotspots.

### Study area

**Fig. 1.**
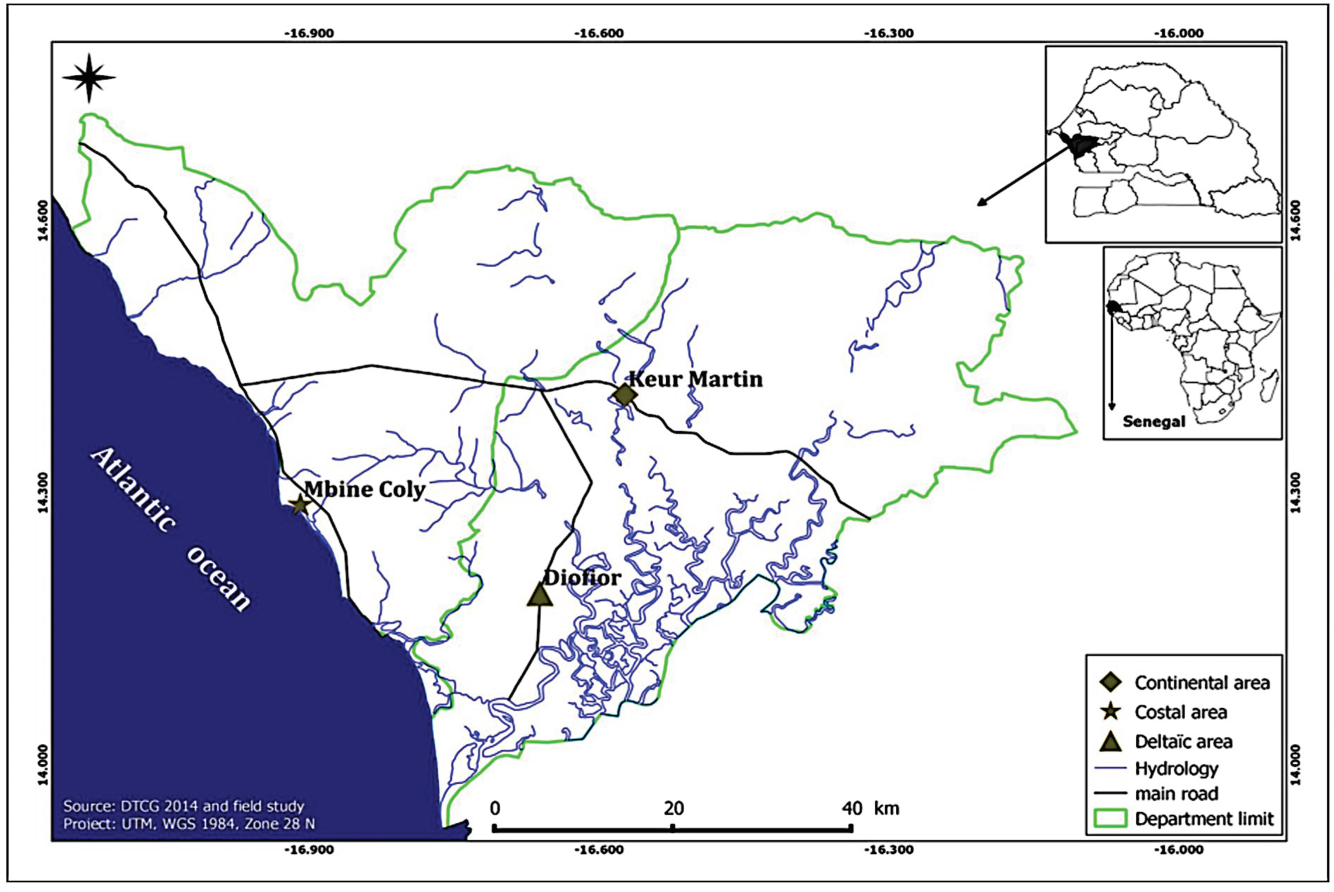
Study area in the central Senegal.

This study was conducted in 3 sites located, respectively, in the coastal, deltaic, and inland (continental) areas in the center-west of Senegal spanning the administrative departments of Mbour and Fatick. The village of Mbind Coly (14°17ʹ10″N; 16°54ʹ30″W) is located in the coastal estuary of Mbour department and is surrounded by a mangrove swamp fed by rainwater and tides. The village of Diofior (13°58ʹ41″N; 16°45ʹ45″W) is located in the deltaic area where the Saloum River flows into the Atlantic Ocean. The village of Keur Martin (14°24ʹ29″N; 16°34ʹ29″W) is located in the western mainland in the department of Fatick and is characterized by the presence of saline soils (Fig. 1). Detailed description of the 3 study sites and areas are reported in Ndiaye et al. (2020) and in Sy et al. (2018).

### Mosquito sampling and processing

The dynamics of malaria vector populations were monitored in each of the study villages using overnight human landing catches (HLC) and Pyrethrum Spray Catches (PSC) in the morning. HLC were performed between 8 PM and 6 AM for 2 consecutive nights in 3 randomly selected houses, where 2 collectors catch landing host seeking, 1 indoor, and another outdoor distant at least 10 m apart from each another. PSC were performed early in the morning, after the second overnight HLC, at least 30 up to 40 randomly selected rooms per village with 1 room per house. Collections were carried out in July, September, October, and December 2017; then in April, May, July, August, September, October, and December 2018. The use of long-lasting insecticide-treated nets (LLINs) by people in the PSC-selected rooms was also reported in a survey form to determine LLINs coverage among the sampled rooms.

### Field and laboratory processing

Collected mosquitoes were morphologically identified to genus level, and Anopheles were subsequently identified to species level using morphological keys (Gillies and Coetzee 1987; Gillies and De Meillon 1968). All mosquito specimens were individually stored in numbered 1.5-ml Eppendorf tubes containing silicagel until laboratory processing. For each collection, 30 randomly sampled female specimens of *An. gambiae* s.l. caught using HLC were dissected to determine the parity rate. The heads and thoraxes of host-seeking females were screened to detect *Plasmodium falciparum* infection using the qPCR TaqMan Assay Method described by Bass et al. (2008). *Anopheles gambiae* s.l. sibling species were discriminated by the PCR method described by Wilkins et al. (2006).

## Data analysis

### Measured parameters

The human-biting rate (HBR) was calculated as the ratio of the total number of females of each vector species collected by HLC to the total person-nights for a given collection period. The parity rate was estimated as the proportion parous from the total number of specimens dissected. The indoor resting density was defined as the number of mosquitoes per room collected by PSC. The sporozoite rate was calculated as the proportion of the total number of mosquitoes infected with *P. falciparum*. The entomological inoculation rate (EIR) was calculated as the product of the HBR and the sporozoite rate. LLIN coverage was calculated as the ratio of the number of rooms with nets used to the number of rooms visited during the PSC collection.

### Statistical analysis

All parameters measured were computed and analyzed using the RStudio and SPSS 26 Statistics software. Data were compared with the Pearson chi-square or Fisher exact tests, Spearman correlation, Kruskal-Wallis test, Friedman ANOVA (non-parametric), or a binomial generalized linear model as applicable, with the statistical significance threshold in all cases set at *P* value ≤0.05.

## Results

### Mosquito densities and species composition

Overall, 3,684 anopheline specimens were collected during the study period, of which 97% (3,575) were *An. gambiae* s.l., 0.6% (22) were *An. funestus*, and 2.4% (87) were *Anopheles pharoensis*. The molecular identification of a sub-sample of 1,877 specimens of *An. gambiae* s.l., including 829 and 1,048 randomly selected specimens respectively from HLC and PSC, revealed the predominance in the study area of *An. arabiensis* with 68.7% (1,290/1,877) and *An. melas* with 28.8% (541/1,877) followed by *An. coluzzii* with 2.13% (40/1,877). During the study period *An. gambiae* (0.27%, 5/1,877) and *coluzzii-gambiae* hybrids (0.05%, 1/1,877) representing 0.3% (6/1,877) of the sub-sample, were the less common members of the complex encountered in the study sites (Table 1). Overall, the species composition was significantly different among the 3 study sites (Pearson’s chi-squared test, X-squared = 30.4, *P*-value = 0.0002). However, the proportions of the 2 most common species were comparable among sites (Pearson’s chi-squared test, X-squared = 0.06, *P*-value = 0.97).

**Table 1.** Distribution of *Anopheles gambiae* sibling species by villages

Espèces	Diofior (Delta)		Keur Martin (inland)		Mbine Coly (Coastal)		Total	
	*N*	%	*N*	%	*N*	%	*N*	%
*An. gambiae*	0	0	1	0.08	4	1	5	0.27
*An. arabiensis*	102	68	920	69.43	268	66.67	1,290	68.73
*An. coluzzii*	5	3.33	20	1.51	15	3.73	40	2.13
*An. melas*	42	28	384	28.98	115	28.60	541	28.82
Hybrid *coluzzii-gambiae*	1	0.67	0	0	0	0	1	0.05
**Total général**	**150**	**100**	**1,325**	**100**	**402**	**100**	**1,877**	**100**

### Resting and biting behaviors of *An. gambiae* s.l. populations

Overall, the resting densities (from PSC) of *An. gambiae* s.l. were at least 2 times higher in the inland area of Keur Martin than in both the deltaic area of Diofior and the coastal area of Mbine Coly. Indoor Resting Density (IRD) of *An. gambiae* s.l. was significantly lower in Diofior than in Mbine Coly and Keur Martin (Friedman chisq = 11.29, *P* = 0.004). IRD varied seasonally and was the highest in September and October, which coincided with the end of the rainy season in all study sites over the 2 years the study was conducted. Thus, in Keur Martin a peak of 5.6 and 7.87 females/room were recorded in September 2017 and September 2018, respectively. In Diofior and Mbine Coly the peaks of IRD were observed in September and October in both years (Table 2).

**Table 2.** Seasonal variation of IRD of *Anopheles gambiae* s.l. population

Month-year	Diofior (Delta)		Keur Martin (inland)		Mbine Coly (Coastal)	
	Collected	IRD	Collected	IRD	Collected	IRD
Jul-2017	0	0.00	32	1.07	22	0.73
Sept-2017	3	0.10	168	5.60	83	2.77
Oct-2017	6	0.20	46	1.53	55	1.83
Nov-2017	0	0.00	0	0.00	0	0.00
Dec-2017	3	0.10	8	0.27	11	0.37
Feb-2018	0	0.00	0	0.00	0	0.00
Apr-2018	0	0.00	0	0.00	0	0.00
Jul-2018	9	0.23	16	0.53	28	0.93
Aug–Sept 2018	5	0.13	236	7.87	19	0.63
Oct-2018	29	0.73	102	3.40	93	3.10
Dec-2018	4	0.10	67	2.23	3	0.10
Total	59	0.24	675	2.05	314	0.95

IRD = indoor resting density.

**Fig. 2.**
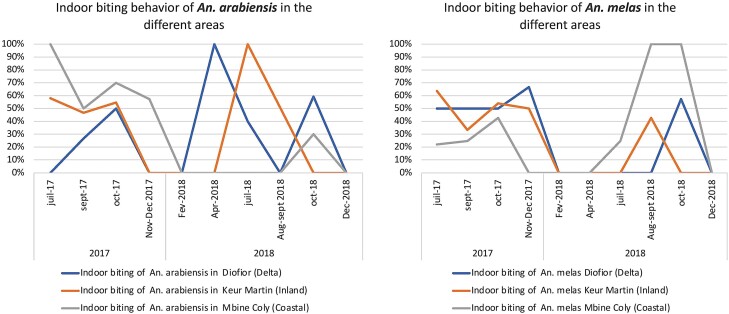
Indoor biting behavior of *Anopheles arabiensis* and *Anopheles melas* in the different study sites.

The endophagic rates of *An. gambiae* s.l. females were comparable in all the studied sites with 49% in both Diofior and Keur Martin and 45% in Mbine Coly (Table 3). The human-biting rate of *An. gambiae* s.l. was the highest in September and October during each year of study. During these 2 months (September and October) no significant difference was noted in HBR among sites, with 4.92 bites per person per night (b/p/n) in the mainland area of Keur Martin, 0.51 b/p/n in Diofior and 0.67 b/p/n in Mbine coly (Friedman chisq = 3.93; *P* = 0.14). Similarly, no significant difference was found in indoor biting proportion (Friedman chisq = 0.80; *P* = 0.67) (Table 3). Overall, during the 2 years of survey, the endophagic rates of both *An. arabiensis* and *An. melas* varied in the same way in the 3 study sites with peaks observed in September and October. From January to June, corresponding to the dry season, only *An. arabiensis* showed endophagic rates of 100% (April 2018) (Fig. 2).

**Table 3.** Seasonal variation of human-biting rates and endophagous rates of *Anopheles gambiae* s.l

Month-year	Diofior (Delta)			Keur Martin (inland)			Mbine Coly (Coastal)		
	Caught	HBR	Indoor (%)	Caught	HBR	Indoor (%)	Caught	HBR	Indoor (%)
Jul-2017	6	0.50	50	37	3.08	59	10	0.83	30
Sept-2017	26	2.17	38	149	12.42	39	37	3.08	49
Oct-2017	10	0.83	50	71	5.92	54	17	1.42	59
Nov–Dec 2017	3	0.25	67	7	0.58	43	7	0.58	57
Fev-2018	0	0.00	0	0	0.00	0	0	0.00	0
Apr-2018	2	0.17	100	0	0.00	0	0	0.00	0
Jul-2018	5^*^	0.21	40	4	0.33	0	4	0.33	25
Aug–Sept 2018	3^*^	0.13	0	382	31.83	50	2	0.17	50
Oct-2018	36^*^	1.50	58	0	0.00	0	11	0.92	27
Dec-2018	0^*^	0.00	0	0	0.00	0	0	0.00	0
Total	91	0.51	49	650	4.92	49	88	0.67	45

^*^From July 2018 two villages are monitored in Diofior and 24 men were used for the 4 nights of human landing catches.

Among the study sites, significant difference was observed in LLIN coverage with 70% in Mbine Coly, 91% in Keur Martin, and 95% in Diofior (chi-squared test, X-squared = 6.24, 2 degrees of freedom, *P* = 0.044) (Table 4).

**Table 4.** LLINs coverage rate in selected PSC rooms in the different sites

Locality	Number of visited houseold	Number of LLINs used	LLIN coverage rate (%)
Mbine Coly	315	222	70
Keur Martin	696	630	91
Diofior	59	56	95

### Parity rates of *An. gambiae* s.l. females

The parity rate of *An. gambiae* s.l. females were 38.9% in the inland area of Keur martin, 56.6% in the coastal zone of Mbine Coly, and 59.6% in the deltaic zone of Diofior. Using a generalized linear binomial model initially including collection month, location, species (*An. arabiensis* or *An. melas*), and a location × species interaction term, only location was significant in the minimal model. The parity rate was significantly lower in Keur Martin than in the reference location Mbine Coly (*P* = 0.023), but Diofior was not significantly different (*P* = 0.30) (Table 5). In all the study sites, *An. arabiensis* displayed the highest parity rate compared to *An. melas* except in the coastal site of Mbine coly where the GLM—species model revealed no significant difference in the parity rate between the 2 species (chi-squared = 5.54; df = 2, *P* = 0.063) (Table 6).

**Table 5.** Seasonal variation of Parity Rate of females of *Anopheles gambiae* s.l caught by HLC

Month-year	Diofior (Delta)			Keur Martin (inland)			Mbine Coly (Coastal)		
	Dissected	Parous	*P* (%)	Dissected	Parous	*P* (%)	Dissected	Parous	*P* (%)
July-2017	3	2	66.6	24	12	50	5	4	80
Sept-2017	16	10	62.5	42	14	33.3	22	15	68.1
Oct-2017	8	3	37.5	37	10	27	14	6	42.8
Nov–Dec 2017	2	1	50	4	2	50	3	2	66.6
Feb-2018	0	0	0	0	0	0	0	0	0
Apr-2018	0	0	0	0	0	0	0	0	0
July-2018	1	0	0	0	0	0	3	3	100
Aug–Sept 2018	2	1	50	100	49	49	2	1	50
Oct-2018	19	13	68.4	0	0	0	11	3	27.2
Dec-2018	0	0	0	0	0	0	0	0	0
Total	52	31	59.6	213	83	38.9	60	34	56.6

**Table 6.** Seasonal variation of Parity Rate of females of *Anopheles arabiensis* and *Anopheles mela*

Month-year	Diofior (Delta)				Keur Martin (inland)				Mbine Coly (Coastal)				TOTAL			
	An. arabiensis		An. melas		An. arabiensis		An. melas		An. arabiensis		An. melas		An. arabiensis		An. melas	
	Dissected	*P*%	Dissected	*P*%	Dissected	*P*%	Dissected	*P*%	Dissected	*P*%	Dissected	*P*%	Dissected	*P*%	Dissected	*P*%
July-2017	0	0	3	67	18	50	6	50	1	0	4	100	19	47	13	69
Sept-2017	8	63	7	57	16	31	26	35	18	67	4	75	42	52	37	43
Oct-2017	2	50	6	33	7	0	30	33	8	50	6	33	17	29	42	33
Nov–Dec 2017	0	0	2	50	0	0	4	50	3	67	0	0	3	67	6	50
Feb-2018	0	0	0	0	0	0	0	0	0	0	0	0	0	0	0	0
Apr-2018	0	0	0	0	0	0	0	0	0	0	0	0	0	0	0	0
July-2018	1	0	0	0	0	0	0	0	0	0	3	100	1	0	3	100
Aug–Sept 2018	1	100	1	0	93	45	5	40	1	0	1	100	95	45	7	43
Oct-2018	14	79	4	50	0	0	0	0	10	20	1	100	24	54	5	60
Dec-2018	0	0	0	0	0	0	0	0	0	0	0	0	0	0	0	0
Total	26	69	23	48	134	42	71	37	41	24	19	74	201	42	113	45

### Sporozoite infection and EIR

The screening of *Plasmodium* infection suggests that *An. arabiensis* and *An. melas* are involved in the residual malaria transmission, with the respective infection rates of 1.39% (*N* = 8) and 0.41% (*N* = 1). No significant difference was observed between species (Fisher exact *P* = 0.29) nor between study sites with species pooled (Fisher exact *P* = 0.87). Infected *An. arabiensis* females were found in Keur Martin (inland area) and Diofior (deltaic area) with the respective infection rates of 1.52% and 1.85%. *Anopheles melas* was found infected only in the inland area (0.54%) (Table 7). No infection was found in the coastal area for both species.

**Table 7.** Local and seasonal variation of sporozoite infection rates of females of *Anopheles arabiensis* and *Anopheles melas* caught by HLC

Month-year	Diofior (Delta)						Keur Martin (inland)						Mbine Coly (Coastal)						TOTAL					
	An. arabiensis			An. melas			An. arabiensis			An. melas			An. arabiensis			An. melas			An. arabiensis			An. melas		
	tested	Positive	CSPR (%)	tested	Positive	CSPR (%)	tested	Positive	CSPR (%)	tested	Positive	CSPR (%)	tested	Positive	CSPR (%)	tested	Positive	CSPR (%)	tested	Positive	CSPR (%)	tested	Positive	CSPR (%)
July-2017	0	0	0	6	0	0	26	0	0	11	0	0	1	0	0	9	0	0	27	0	0	26	0	0
Sept-2017	15	0	0	10	0	0	60	0	0	89	0	0	32	0	0	4	0	0	107	0	0	103	0	0
Oct-2017	4	0	0	6	0	0	11	0	0	59	0	0	10	0	0	7	0	0	25	0	0	72	0	0
Nov–Dec 2017	0	0	0	3	0	0	1	0	0	6	** 1**	16.67	7	0	0	0	0	0	8	0	0	9	1	** 11.11**
** Sub total**	** 19**	** 0**	0	** 25**	** 0**	0	** 98**	** 0**	0	** 165**	1	0.61	** 50**	** 0**	0	** 20**	** 0**	0	** 167**	0	0	210	1	** 0.48**
Feb-2018	0	0	0	0	0	0	0	0	0	0	0	0	0	0	0	0	0	0	0	0	0	0	0	0
Apr-2018	2	0	0	0	0	0	0	0	0	0	0	0	0	0	0	0	0	0	2	0	0	0	0	0
July-2018	5	0	0	0	0	0	4	0	0	0	0	0	0	0	0	4	0	0	9	0	0	4	0	0
Aug–Sept 2018	1	0	0	1	0	0	358	** 7**	1.96	21	0	0	1	0	0	1	0	0	360	7	** 1.94**	23	0	0
Oct-2018	27	** 1**	3.70	7	0	0	0	0	0	0	0	0	10	0	0	1	0	0	37	1	** 2.70**	8	0	0
Dec-2018	0	0	0	0	0	0	0	0	0	0	0	0	0	0	0	0	0	0	0	0	0	0	0	0
** Sub total**	35	0	0	8	0	0	362	0	0	21	0	0	11		0	6	0	0	** 408**	0	0	35	0	0
** Total**	** 54**	** 1**	** 1.85**	** 33**	** 0**	** 0**	** 460**	** 7**	** 1.52**	** 186**	** 1**	** 0.54**	** 61**	** 0**	** 0**	** 26**	** 0**	** 0**	** 575**	** 8**	** 1.39**	** 245**	** 1**	** 0.41**

**Fig. 3.**
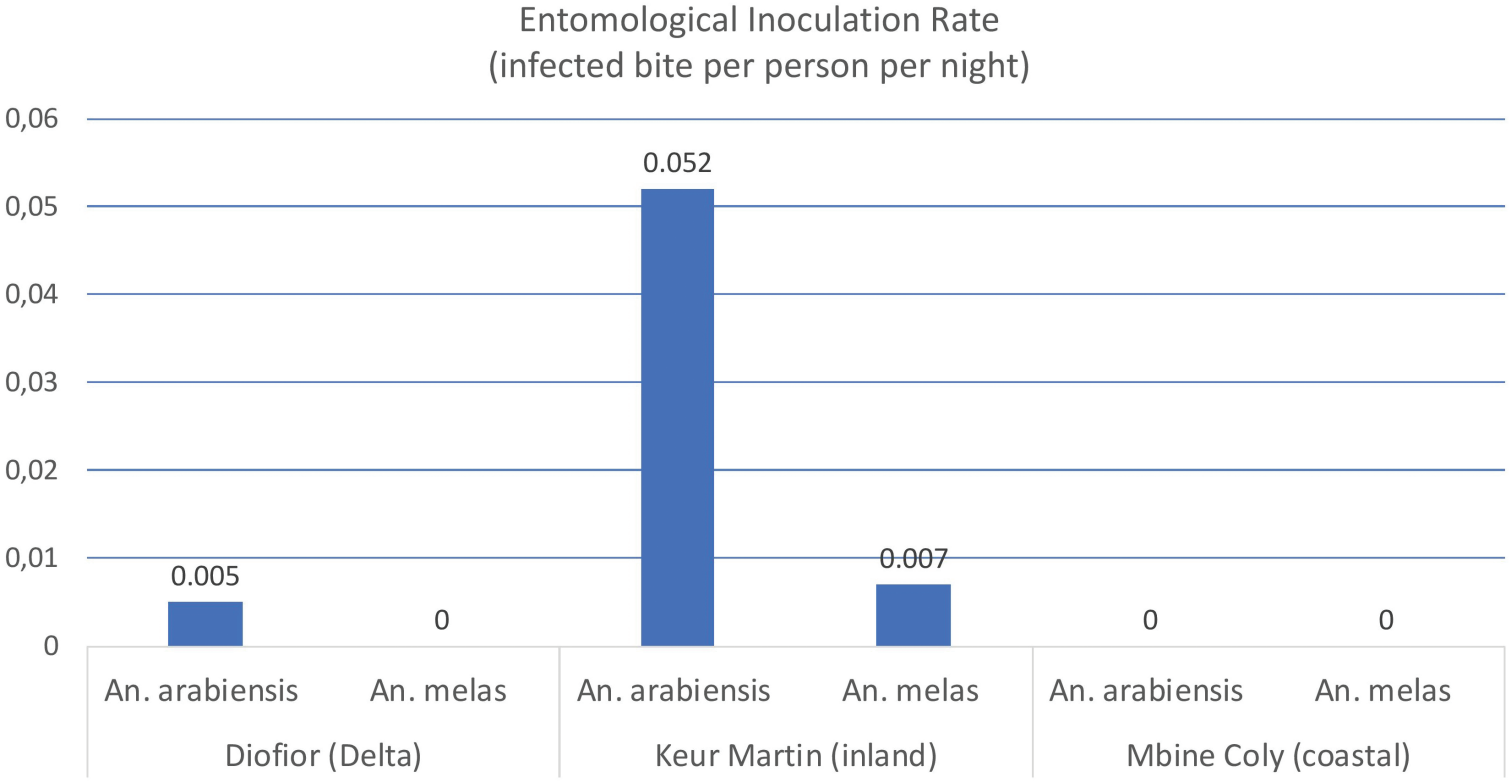
EIRs of *Anopheles arabiensis* and *Anopheles melas* in the different sites.

Overall, in the study area, despite the presence of *An. coluzzii* and *An. gambiae*, only *An. arabiensis* and *An. melas* seem to play a role in malaria transmission, being the only species found infected with respectively EIR of 0.018 and 0.002 ib/p/n, corresponding to an annual infected bites of 6.57 ib/p/year for *An. arabiensis* and 0.73 ib/p/year for *An. melas*. Both species were found infected in the same locality at the same time only in the inland area of Keur Martin (Table 7). No *Plasmodium* infection was found in the coastal area of Mbine Coly, whereas in the deltaic area of Diofior, only *An. arabiensis* was found infected with an EIR of 0.0055 ib/p/n (or 2 ib/p/year [Fig. 3]).

## Discussion

This study was conducted over 2 consecutive years in 2017 and 2018 and confirmed the predominance of *An. gambiae* s.l. representing 97% of the anopeline fauna with minor presence of other species, *An. funestus* and *An. pharoensis*. Indeed, the *An. gambiae* s.l. complex contains the major malaria vectors among the 22 species described nationwide (Diagne et al. 1994, Coetzee et al. 2000). The use of molecular approaches on morphologically identified *An. gambiae* s.l. revealed the presence of 4 members of the gambiae complex: *An. arabiensis*, *An. melas, An. gambiae*, and *An. coluzzi,* and the presence of *gambiae/coluzzii* hybrids. In the study area, *An. arabiensis* and *An. melas* were the 2 most abundant species of the gambiae complex. *Anopheles arabiensis* is known as a predominant malaria vector over all the country specially in the dried and arid environments, except the south and south–east part of Senegal (Dukeen and Omer 1986, Faye et al. 1995, Lemasson et al. 1997). The presence of *An. melas* was reported in brackish water resulting from the mixture of sea water with the freshwater of the Saloum River in the deltaic area where it flows into the ocean, known to be provide preferred breeding sites (Bryan et al. 1987, Faye et al. 1994). The IRD of *An. gambiae* s.l. was relatively more important in the inland area of Keur Martin than in the 2 other areas (deltaic and coastal areas). In contrast to the other study sites, Keur Martin village is characterized by the presence of hydromorphic and halomorphic soils, which in addition to their capacity to retain water over a long period of time, are characterized by the presence of surface brackish water bodies, suitable for the development of *An. arabiensis* and *An. melas* depending on the level of salinity (Ndiaye et al. 2020). In all the sites, the exophagy rate of *An. gambiae* s.l females have been relatively important, this situation could be explained by the predominance of *An. arabiensis*, accounting for 68.72% of the anopheline fauna in the area and known for its behavioral plasticity, being more exophilic in some areas compared to its sibling (Mahande et al. 2007). Furthermore, the high coverage of LLINs, following the LLIN mass campaign implemented by the PNLP in 2016 across the study area could suggest additional pressure and which could lead to an induced exophilic behavior due to the repellent effect of pyrethroid, the class of insecticide used for net impregnation, as previously shown in an experimental study (Darriet et al. 2002, PNLP 2016). The parity rate of *An. gambiae* s.l. was the lowest in the inland site of Keur martin (38.9%) and was comparable with the rates (25–33%) previously reported from the mangrove area in the Gambia (Bryan et al. 1987) and from Casamance (47%) (Faye et al. 1994). The high level of pyrethroid-impregnated LLINs coverage in the studied houses of Keur Martin (>90%) could explain the reduced longevity of the studied populations of *An. gambiae* s.l.

Only *An. arabiensis* and *An. melas* were found infected in the study area, this finding is in line with recent studies carried out in the central Senegal (Sy et al. 2018).

Generally, human populations are at high risk of being infected by malaria local vector species, especially during the second half of the night when they were not effectively using the LLINs. LLINs mass distribution campaign and IRS are the 2 main malaria vector control strategies in Senegal and sub-Saharan Africa generally (Dione 2014, Sy et al. 2019, PMI 2020) with the main goals to reduced host-vector contact and vector population longevity. Several authors have reported the possible involvement of *An. arabiensis*, *An. melas*, and *An. coluzzii* species, possibly playing a main role in malaria transmission in the study area here (Hamon et al. 1963, Mahande et al. 2007, Sy et al. 2018). The absence of infected females of *An. coluzzii* during the current study could be explained by the low sample representativity of this species during the study period. The average EIR for *An. arabiensis* and *An. melas* almost 7 time higher for the first species than the latter without significant difference due to the low numbers of *An. melas*, being nevertheless lower than reported previously in same the area with 13.14 infective bites per person per year (Sy et al. 2018). This low level of transmission as observed here is probably related to a variety of malaria control interventions implemented by the NMCP in this area. Indeed, a SMC in children under 10 years of age was implemented in the area between 2008 and 2011, followed by 2 targeted indoor residual spraying campaigns with pirimiphos-methyl in 2013 and 2014 (Cissé et al. 2016, Sy et al. 2019).

## Conclusion

This study demonstrated the predominance and the involvement of both *An. arabienis* and *An. melas* in maintaining malaria in hotspot found in an overall low malaria transmission in the central Senegal. The findings were generated on infection using a sensitive qPCR approach, which allows the detection of low plasmodial infection in mosquitoes, suitable for areas of low malaria transmission. The data generated will be shared with the PNLP and will certainly allow to better tailor the monitoring of malaria in this area, eligible for malaria elimination. Subsequent investigations on insecticide phenotypic resistance and intensity for these 2 species in the area, are needed to better complement evidence to better select and target vector control strategies to drive toward the goal of malaria elimination as aimed by the NMCP in eligible areas.
